# UCHL1 Promotes Epithelial–Mesenchymal Transition Through E-Cadherin and N-Cadherin Switch in Endometrial Cancer

**DOI:** 10.3390/biomedicines14102168

**Published:** 2026-09-24

**Authors:** Azusa Kobayashi, Kohshiro Nakao, Naoki Inoue, Tae Oike, Sadatomo Ikeda, Takashi Hirakawa, Orie Nakamura, Akira Iwase

**Affiliations:** 1 Department of Obstetrics and Gynecology, Gunma University Graduate School of Medicine, 3-39-22, Showa-Machi, Maebashi 3718511, Gunma, Japan; a.koba.haruchagu@gmail.com (A.K.); konakao04@gmail.com (K.N.); naokiinoue1987@gmail.com (N.I.); thira@gunma-u.ac.jp (T.H.); nakamura-orie@hro.or.jp (O.N.); 2 Department of Research and Development Division, Kishokai Medical Corporation, 2-4-15, Naka-ku Nishiki, Nagoya 4600003, Aichi, Japan

**Keywords:** endometrial cancer, UCHL1, epithelial–mesenchymal transition, cadherins

## Abstract

**Objective:** Endometrial cancer (EC) is one of the most common cancers in women, and its incidence is increasing worldwide. The prognosis of recurrent and advanced cancer remains poor because treatment options are limited. Ubiquitin C-terminal hydrolase-L1 (UCHL1) is associated with the progression of several cancers including EC. In this study, we analyzed the function of UCHL1 in EC progression. **Methods:** UCHL1 expression in the serum of patients with EC was measured using an enzyme-linked immunosorbent assay. Changes in cell migration and invasion were analyzed in UCHL1-overexpressing or UCHL1-knockdown cells (UCHL1-OE/UCHL1-KD) using wound-healing and transwell assays. Messenger RNA expression of epithelial–mesenchymal transition (EMT)-related genes in each cell was detected using quantitative real-time polymerase chain reaction (qRT-PCR). UCHL1 and EMT marker proteins were detected with Western blotting and an immunofluorescence assay (IFA). **Results:** Serum UCHL1 expression was significantly elevated in patients with metastases. Wound-healing assays and transwell assays showed that UCHL1 could promote cell migration and invasion. These effects were suppressed by UCHL1 knockdown and inhibition. UCHL1 overexpression modulated the expressions of EMT-related molecules in EC cells at the mRNA and protein levels. Furthermore, IFA analysis showed that changes in EMT-related molecules were consistent with lesions of UCHL1 expression. **Conclusions:** This study revealed that UCHL1 could contribute to promoting cell migration and invasion. These results may be associated with EMT regulation in EC cells.

## 1. Introduction

Endometrial cancer (EC) is the fifth most common cancer in women, and its incidence is increasing worldwide. A total of 420,242 new cases and 97,704 deaths due to EC occurred in 2022 [1]. The incidence of EC in Japan is consistent with the global trend [2]. Most patients with EC are diagnosed at an early stage and have a favorable prognosis with surgery and appropriate adjuvant treatment. In EC staging systems, a concept based on The Cancer Genome Atlas (TCGA) classification has been proposed, and EC management is changing accordingly. There are several treatment options for patients with advanced disease or disease recurrence. In locally advanced cases, surgical resection remains the primary treatment option if macroscopically complete resection is feasible. Furthermore, the efficacies of radical radiotherapy and neoadjuvant chemotherapy have been demonstrated [3,4,5]. In addition to the conventional carboplatin paclitaxel therapy, new drugs are now available for advanced or recurrent EC. The prognosis of these cases improves with a combination of carboplatin paclitaxel and pembrolizumab or durvalumab [6,7]. However, the options for effective treatment remain limited when response to first-line treatment is poor [4,5]. Even when the disease responds to treatment, patients require long-term drug therapy, which exposes them to physical, psychological, and social stressors. It is important to understand the biological mechanisms underlying these poor prognoses to develop effective treatments and diagnostic approaches.

The epithelial–mesenchymal transition (EMT) is a phenomenon in which cells lose their epithelial properties and acquire mesenchymal abilities. The EMT plays a crucial role in embryonic development, wound healing, and tumor progression [8,9,10], and promotes tumor cell invasion, migration, and metastasis during tumor progression. Several reports have indicated that the EMT results in poor prognosis in patients with EC [11,12,13]. A previous study reported that upregulation of the EMT (loss of E-cadherin and expression of EMT transcription factors) was observed in advanced EC tissues and in those with positive lymph node metastasis [11]. Furthermore, high expression of mesenchymal markers has been reported in EC with positive lymph node metastasis [12]. These results suggest that the EMT is a biological mechanism that could be a therapeutic target for EC with advanced disease or recurrence status.

Post-translational modification of proteins by ubiquitination is a major molecular mechanism that maintains homeostasis in organisms. Deubiquitinating enzymes (DUBs) remove ubiquitin from ubiquitinated proteins and play important role in ubiquitination. Ubiquitin C-terminal hydrolase-L1 (UCHL1), a DUB, hydrolyzes the ubiquitinated protein isopeptide bond and promotes target protein stabilization by preventing its proteasomal degradation. UCHL1 is highly expressed in neurons and gonads, and is one of the causative molecules of Parkinson’s disease [14]. UCHL1 promotes cancer proliferation, invasion, migration, and metastasis by stabilizing cancer-associated molecules in cancer cells [14,15]. Associations between UCHL1 and cancer progression have been reported in several cancers including lung, breast, bladder, ovarian, and uterine cervical cancers [16,17,18,19,20,21,22,23,24,25,26]. Research on breast cancer has suggested UCHL1 stabilizes the epidermal growth factor receptor (EGFR) by deubiquitination and may regulate estrogen receptor expression [18]. Furthermore, a study showed that UCHL1 promotes tumor metastasis by stabilizing HIF1α [26]. Studies have also reported that UCHL1 regulates the EMT in cancer cells [17,19,20,21,22,23,24,25]; UCHL1 stabilizes pyruvate kinase M2, which suppresses CDH1 transcription, thereby promoting the EMT in bladder cancer [19]; and UCHL1 promotes the EMT in breast cancer cells by stabilizing TGF-β receptor I and SMAD [24].

In uterine EC, it was suggested that UCHL1 regulates cell proliferation and the cell cycle of uterine serous carcinoma cells, and may be involved in tumor progression [27]. A previous study showed that UCHL1 expression in EC tissues is associated with poor prognostic factors. The prognosis of patients with high UCHL1 expression was poorer than that of those with low UCHL1 expression. Furthermore, UCHL1 expression in metastasis regions may be associated with poor prognosis [28]. However, the functions of UCHL1 in EC remain unknown. In this study, we analyzed the association between UCHL1 expression and EC progression.

## 2. Materials and Methods

### 2.1. Patient Specimens

Sera were sampled from 149 patients with EC before surgery. Patient information, EC stage, and histological type were collected from medical records. This study was approved by the Institutional Review Board of the Gunma University Hospital (IRB, approval no. HS2020-207). Serum UCHL1 levels were measured using a Human UCHL1-ELISA Kit (KE00211, Proteintech Japan, Tokyo, Japan), according to the manufacturer’s protocol.

### 2.2. Cell Culture

Human EC cell line HEC1A was purchased from American Type Culture Collection (Manassas, VA, USA). Human EC cell lines Ishikawa and HEC251 were purchased from Japanese Collection of Research Bioresources (JCRB) cell bank (Tokyo, Japan). Cells were cultured in Dulbecco’s Modified Eagle Medium (DMEM) (16791-55, Nacalai Tesque, Kyoto, Japan) supplemented with 10% fetal bovine serum (FBS) and penicillin streptomycin solution (168-23191, FUJIFILM Wako, Osaka, Japan) at 37 °C in 5% CO_2_. UCHL1 expression level is low in HEC1A and Ishikawa and high in HEC251. The UCHL1 expression levels in these cell lines are provided in the Supplementary Figure S1. The cell lines were used in the experiments within 10 passages from the start of culture.

### 2.3. Construction of the UCHL1 Expression Vector and Generation of UCHL1-Overexpressing Stable Clones

The human UCHL1 coding sequence (CDS) was amplified using PCR from cDNA derived from an EC patient and cloned into pBApo-CMV Neo DNA (3240, TaKaRa, Shiga, Japan) with restriction enzymes and T4 DNA Ligase (M0202S, NEB Japan, Tokyo, Japan). The UCHL1 expression vector was transfected into HEC1A cells using Xfect^TM^ Transfection Reagent (631317, TaKaRa, Shiga, Japan) according to the manufacture’s protocol. The cells were selected with 100 μg/mL G418 (08973-01, nacalai tesque, Kyoto, Japan) to generate UCHL1-overexpressing stable clone. Based on the UCHL1 expression vector, UCHL1 tagged with 3× FLAG dsDNA was constructed and cloned to pBApo-EF1α Pur DNA (3244, TaKaRa, Shiga, Japan). The Ishikawa cells were transfected with the UCHL1-3× FLAG expression vector. A UCHL1-3× FLAG-overexpressing stable clone was generated with 1 μg/mL puromycin (160-23151, FUJIFILM Wako, Osaka, Japan). Cell lines transfected with the empty vector were used as controls.

### 2.4. Small Interfering RNA (siRNA) Transfection and Treatment with a UCHL1 Inhibitor

An siRNA targeting UCHL1 (siUCHL1) was designed using siDirect (https://sidirect2.rnai.jp/, accessed on 5 April 2024). The scrambled sequence of siUCHL1 was used as a negative control (siNC). Both were generated by Eurofins Genomics. HEC251 cells were transfected with 20 nM siRNA using Lipofectamine^®^ RNAiMAX Reagent (13778075, ThermoFisher SCIENTIFIC, Waltham, MA, USA) according to the protocol. The cells were used for each experiment 72 h after transfection. For the in vitro experiments, #2 siRNA for UCHL1 was used because of its higher knockdown efficiency. The siRNA sequences are listed in Supplementary Table S1.

### 2.5. Protein Isolation and Western Blotting Analysis

The cells were extracted and lysed using a RIPA buffer (50 mM Tris HCl pH 7.6, 150 mM NaCl, 1% NP-40, 0.1% sodium dodecyl sulfate (SDS), 0.5% sodium deoxycholate, and 10 mM sodium fluoride) containing a protein inhibitor cocktail (03969-21, Nacalai Tesque, Kyoto, Japan) and 1 mM PMSF (162-12182, FUJIFILM Wako, Osaka, Japan). After sonication and centrifugation at 15,000 rpm for 15 min, the protein concentration was calculated using a BCA protein assay kit (297-73101, FUJIFILM Wako, Osaka, Japan). The samples were adjusted to 1 μg/μL by mixing with 5× Laemmli buffer. Proteins were separated using SDS polyacrylamide gel electrophoresis and transferred to polyvinylidene difluoride membranes. The membranes were incubated with 5% skim milk in TBS-T for 1–1.5 h at room temperature and subsequently probed with the primary antibodies at 4 °C overnight. After incubation with secondary antibodies for 1 h at room temperature, protein signals were detected using enhanced chemiluminescence method.

Primary antibodies were anti-UCHL1, anti-E-cadherin, anti-N-cadherin and anti-Vimentin (14730-1-AP, 20874-1-AP, 22018-1-AP and 10366-1-AP, Proteintech Japan, Tokyo, Japan). The secondary antibody was HRP-conjugated Goat Anti-Rabbit IgG (SA00001-2, Proteintech Japan, Tokyo, Japan). HRP-conjugated GAPDH Monoclonal antibody (HRP-60004, Proteintech Japan, Tokyo, Japan) was used as the antibody for internal control.

### 2.6. Wound-Healing Assay

UCHL1-overexpressing (UCHL1-OE) HEC1A and Ishikawa cells were cultured in 6-well plates (5 × 10^5^ cells/well) until they reached 80% confluence. The center of each well was scratched with a 200 μL tip. In UCHL1-knockdown (UCHL1-KD) HEC251 cells, 2 × 10^5^ cells were cultured in each well of a 6-well plate and scratched 72 h after siRNA transfection. The UCHL1 inhibitor, LDN57444, was added to HEC251 cells (2 × 10^5^ cells/well, 6-well plate) at the same time as cell scratching. The open wound areas were observed every day and calculated as a ratio relative to the area on day 0. The images were analyzed using ImageJ 1.54. The colors of images were changed to black and white to help show the results more clearly.

### 2.7. Cell Transwell Assay

Transwell chambers (CH8-24, KURABO, Okayama, Japan) were placed in 24-well plates and coated with 100 μg/well EHS-gel Basement Membrane Matrix (066-09031, FUJIFILM Wako, Osaka, Japan). UCHL1-OE Ishikawa cells (5 × 10^4^ cells/well) were added to the upper chamber with 100 μL serum-free medium. In the lower wells, 300 μL DMEM containing 10% FBS was added and the cells were incubated for 16 h. The surfaces of the chambers that were in contact with the lower medium were fixed in 70% ethanol and stained with hematoxylin. The numbers of cells that had infiltrated the lower chamber’s surface were counted. HEC251 with UCHL1-knockdown or UCHL1-inhibitor treatment were cultured at 2 × 10^5^ cells/well and incubated for 16 h, and the other protocol was similar to that used with UCHL1-OE cells. The images were analyzed using ImageJ 1.54.

LDN57444 was added to HEC251 cells at the same time with the cell culture into the transwell chamber.

### 2.8. Cell Proliferation Assay

Cell proliferation was analyzed using Cell Counting Kit-8 (CCK-8, 341-07761, DOJINDO, Kumamoto, Japan). UCHL1-OE Ishikawa cells, UCHL1-OE HEC1A cells and control cells (2000 cells/well) were cultured in a 96-well plate. After incubating for 24–96 h, 10 μL CCK-8 solution was added to each well at 24 h intervals and the absorbance at 450 nm was measured 2 h after the addition of CCK-8 solution. UCHL1-KD HEC251 cells (2000 cells/well) were added to a 96-well plate 72 h after transfection of siRNA and analyzed with same method. The mean values of three experiments were calculated under the same conditions.

### 2.9. Quantitative Real-Time PCR (qRT-PCR)

Total RNA from cells was extracted using NucleoSpon^®^ RNA Plus (740984.250, MACHEREY-NAGEL GmbH & Co.KG, Duren, Germany). 1 μg total RNA was subjected to reverse transcription to synthesize single-stranded cDNA using ReverTra Ace^®^ qPCR RT Master Mix (FSQ-201, TOYOBO, Osaka, Japan). qRT-PCR was performed with GeneAce SYBR™ qPCR Mixα Low Rox (316-07693, NIPPON GENE, Tokyo, Japan). Relative mRNA levels of the target gene were quantified using the comparative Ct(2^−ΔΔCT)^ method. The primer sequences are listed in Supplementary Table S2.

### 2.10. Immunofluorescence Assay (IFA)

The cells were cultured overnight on chamber slides (SCS-N04, MATSUNAMI, Osaka, Japan), washed with PBS, and fixed with 4% paraformaldehyde for 15 min at room temperature. After washing with PBS and infiltrating with 0.2% Triton X-100, the cells were incubated with 5% BSA in PBS/0.2% Triton X-100 for 1 h at room temperature. The cells were probed with primary antibodies at 4 °C overnight and subsequently incubated with secondary antibodies for 1 h at room temperature. Counterstaining for cell nuclei was performed with Mounting Medium with DAPI Aqueous, Fluoroshield (ab104139, abcam, Tokyo, Japan) for 5 min. After sealing with a cover glass, the cells were observed using epifluorescence microscopy (40× objective). The images were analyzed using ImageJ 1.54. In each experiment, three randomly selected cells were analyzed by outlining the whole cell area, and the corrected total cell fluorescence (CTCF) was calculated for each cell. The mean CTCF of three cells was used as the representative value for each experiment. The mean values from the three independent experiments were statistically compared.

As primary antibodies, the DYDDDDKtag Monoclonal antibody (66008-4-Ig, Proteintech Japan, Tokyo, Japan) was used for UCHL1-OE Ishikawa cells and, UCHL1 Mouse mAb (60702, Cell Signaling Technology, Tokyo, Japan) was used for UCHL1-KD HEC251 cells. The antibodies used for detecting E-cadherin and N-cadherin were the same as those used for the Western blot analysis. The secondary antibodies used were Goat anti-rabbit IgG FITC Conjugate and, Coralite594-Conjugated Goat Anti-Mouse IgG (SA00003-2, SA00013-3, Proteintech Japan, Tokyo, Japan).

Each experiment (Western blotting, qRT-PCR, in vitro assay, and ICC) was repeated thrice under the same conditions.

### 2.11. Statistical Analysis

All statistical analyses were performed using IBM SPSS Statistics 29.0 (IBM, Armonk, NY, USA). The Mann–Whitney U test was used to compare between groups of patient specimens. Other statistical analyses between the two groups were performed using Student’s *t*-test. The threshold for statistical significance for all analyses was set at *p* < 0.05.

## 3. Results

### 3.1. Increased Serum UCHL1 Levels in Patients with Metastases

A previous study reported that high UCHL1 expression in tissues of patients with EC was associated with a poor prognosis [28]. First, we determine the association between UCHL1 expression in patient serum and metastasis. The patients were classified according to the presence or absence of metastases, including lymph node and distant metastases. The serum UCHL1 level was significantly high in the patients with metastases, suggesting a potential association between UCHL1 and metastasis in EC progression (Figure 1).

### 3.2. UCHL1 Promoted Invasion and Migration of EC Cells

To analyze the effects of UCHL1 on EC cells, we established UCHL1-OE cell lines. UCHL1 expression vectors were transfected into the EC cell lines HEC1A and Ishikawa. Western blotting confirmed UCHL1 expression in both cell lines (Figure 2A). We conducted the experiments and analyzed the effects of UCHL1 expression on cell migration and invasion. In the wound-healing assay, migration was significantly enhanced in the UCHL1-OE HEC1A cells and Ishikawa cells (Figure 2B,C). Furthermore, the transwell assay showed a significant increase in the number of invasive UCHL1-OE Ishikawa cells compared with the control cells (Figure 2D). These results suggest that UCHL1 overexpression in EC cells promotes cell migration and invasion. We performed proliferation assays using UCHL1-OE cells. However, there was no significant differences in the cell proliferation between the UCHL1-OE cells and control cells (Figure S2).

### 3.3. UCHL1-Knockdown- or UCHL1-Inhibitor-Treated EC Cells Showed Reduced Migration and Invasion

To further evaluate the effects of UCHL1 in EC cells, a transient knockdown experiment of UCHL1 was conducted. An siRNA targeting UCHL1 (siUCHL1) or a negative control siRNA (siNC) was transfected into HEC251 cells, which naturally express high levels of UCHL1. Effective UCHL1 knockdown was using Western blotting prior to the experiment (Figure 3A). In the wound-healing assay, the reduction in scratched area was significantly slower in the UCHL1-KD cells compared with the control cells, indicating reduced migration ability (Figure 3B). Furthermore, the invasion assay showed a significant decrease in the number of invasive cells in the UCHL1-KD cells (Figure 3C). In the proliferation assay, proliferation of the UCHL1-KD cells was not significantly suppressed compared with the control cells (Figure S3).

To confirm the effect of the UCHL1 inhibitor, we performed the same experiments after adding the UCHL1 inhibitor LDN57444 to the HEC251 cells. Similar to the knockdown cells, the wound-healing assay showed reduced migration in cells treated with the UCHL1 inhibitor (Figure 3D). In the transwell assay, the inhibitor significantly reduced the invasive ability of the cells (Figure 3E).

Considering the results from the UCHL1-OE cells, it was clarified that UCHL1 regulates cell migration and invasion.

### 3.4. Modulation of UCHL1 Altered EMT-Related Molecules in EC Cells

Based on the in vitro assay results, we verified the association between UCHL1 and EMT markers in the EC cells. The mRNA expression of EMT-related molecules in the UCHL1-OE and UCHL1-KD cells was analyzed using qRT-PCR. In the UCHL1-OE Ishikawa cells, the epithelial marker CDH1 was downregulated, whereas the mesenchymal markers, CDH2 and VIM were upregulated. Significant differences were observed in all results (Figure 4A). In the UCHL1-KD HEC251 cells, CDH2 expression was significantly downregulated, whereas CDH1 was unchanged. VIM expression was significantly increased in UCHL1-KD cells (Figure 4B) In the analysis conducted with an extended knockdown time, CDH1 levels increased significantly in UCHL-KD; however, the VIM expression remained unchanged (Figure S4).

The protein expression of EMT markers was analyzed using Western blotting, which showed that N-cadherin and vimentin were markedly upregulated, whereas E-cadherin was downregulated in the UCHL1-OE Ishikawa cells, consistent with the qPCR results (Figure 5A). In the UCHL1-KD HEC251 cells, N-cadherin expression was significantly reduced, whereas E-cadherin and vimentin levels were unchanged compared with the controls (Figure 5B). IFA analysis showed significantly decreased E-cadherin and increased N-cadherin expression in the UCHL-OE Ishikawa cells, consistent with the Western blotting results. In the UCHL1-KD cells, N-cadherin expression was significantly suppressed, whereas the E-cadherin level was unchanged compared with the siNC controls (Figure 5C).

To further explore the effects of UCHL1 modulation on the EMT, qRT-PCR analysis of EMT-inducing transcription factors (EMT-TFs) was performed using UCHL1-OE Ishikawa cells and UCHL1-KD HEC251 cells. In the UCHL1-OE cells, the mRNA expression levels of SLUG, TWIST1, ZEB1 and ZEB2 were significantly upregulated (Figure 6A). SNAIL in the UCHL1-OE cells was also higher than in the control cells; however, the difference was not significant. In the UCHL1-KD cells, SNAIL, SLUG and TWIST1 mRNA levels were significantly downregulated. Although ZEB1 and ZEB2 expression tended to be suppressed, the results were not statistically significant (Figure 6B). These results showed that UCHL1 may be involved in the regulation of cadherins and EMT-TFs expressions in EC cells.

## 4. Discussion

A previous report has indicated that high UCHL1 mRNA expression in EC tissues correlates with poor prognosis, and that UCHL1 may affect the progression of metastatic lesion [28]. Although these clinical observations imply a role for UCHL1 in tumor progression, its function regarding EC cells remains unclear, as UCHL1 can exert context-dependent effects in cancer. In this study, high UCHL1 expression was observed in the sera of patients with metastases, suggesting a possible involvement of UCHL1 in EC metastasis. To explore its functional role, we examined the effects of UCHL1 in EC cells, and found that UCHL1 promoted the cell migration and invasion. These changes in cell abilities may be related with EMT modulation in EC cells.

One of DUB’s primary roles is removing ubiquitin proteins from target substrates, but its function in the ubiquitin system is very complex [29,30]. UCHL1 contains a catalytic domain that binds to the C-terminus of ubiquitin and cleaves it from protein substrates. It is also known to contribute to the ubiquitination of target proteins as a ubiquitin ligase and monoubiquitin stabilization [14,15]. Poor survival rates and UCHL1 overexpression of tumor cells have been demonstrated in several cancers, such as breast, bladder, ovarian, and uterine cervical cancers [17,19,20,22,28,31,32]. These studies have reported that UCHL1 stabilizes oncoproteins and induces signaling pathways that promote tumor progression. In non-small-cell lung cancer cells, UCHL1 promotes PD-L1 expression in lung cancer cells through Akt signaling regulation [16]. A study on renal cell carcinoma has reported that UCHL1 stabilizes KDM4B, a histone demethylase, thereby deubiquitinating it and promoting vascular endothelial growth factor A (VFGFA) transcription [32]. In contrast, UCHL1 has also been reported to function as a tumor suppressor [30,33,34,35]. Some reports have shown that UCHL1 promotes apoptosis in tumor cells by inhibiting the proteasomal degradation of p53 [33,34]. In a study on nasopharyngeal carcinoma, UCHL1 ligase activity increased K48-linked ubiquitination of Cortactin, a member of the actin-binding protein family, thereby promoting its degradation. This change suppressed tumor cell migration, invasion, and metastasis [35]. These opposing reports on UCHL1 function may be related to the type of cancer cells or target molecules examined. A complex balance may exist between ubiquitin and DUBs in cancer cells to maintain cellular functions.

A study on uterine serous carcinoma indicated that UCHL1 promotes the proliferation of cancer cells [27]. This effect results from the stabilization of cyclin B1 protein by UCHL1, thereby accessing the cell cycle. Our results showed no significant differences in cell proliferation, which was inconsistent with this report. The same report also indicated that UCHL1 was overexpressed in conventional type II EC cell lines. The function and expression of UCHL1 may differ between uterine cancer cells. This will provide clues for elucidating UCHL1 function in EC cells.

Our findings initially demonstrate that UCHL1 promotes the migration and invasion of EC cells, where the regulation of EMT molecules may be involved in these changes. The expression of EMT-related molecules in UCHL1-KD cells partially contradicts this prediction. These results may be unstable because the knockdown method involved transient transfection. Since the UCHL1-OE cells used in these experiments were stable clones, we considered their results to be more reliable. The significant increase in CDH1 was observed in UCHL1-KD cells with extended knockdown time. After UCHL1 knockdown, significant changes in downstream molecules may occur over time. However, the mRNA expression of VIM was not suppressed with extended knockdown time. These results indicate that there is still insufficient evidence to conclude that UCHL1 promotes the EMT.

A cadherin switch was significantly observed in UCHL1-OE cells, characterized by decreased E-cadherin and increased N-cadherin expression. Low expression of E-cadherin induces intercellular adhesion and polarity losses, whereas increased expression of mesenchymal markers enables cell migration. Some reports have indicated that reduced E-cadherin levels due to EMT in breast cancer and EC correlate with poor prognosis [11,13,36]. Several studies examined the relationship between the EMT and DUBs [17,19,22,23,24,25,37]. USP20 has been reported to stabilize SNAI2 and promote the metastatic potential of breast cancer [37]. A study on colorectal cancer has demonstrated that UCHL1 promotes the invasion and migration of cancer cells through β-catenin deubiquitination [25]. In our study, it was revealed that the localization of UCHL1 expression coincided with changes in E-cadherin and N-cadherin using IFA analysis. Furthermore, EMT-TFs’ expression was changed by UCHL1 modulations. These results indicate that UCHL1 may be one of the factors contributing to the cadherin switch.

As is well known, the EMT plays a crucial role in the acquisition of metastatic ability and exhibits similar characteristics in EC [11,12,13]. Our study also showed that UCHL1 expression significantly increased in the sera of patients with metastases. Our finding suggests a possible association between serum UCHL1 levels and metastasis in EC. However, because this study did not include the results of in vivo experiments, whether UCHL1 contributes to the formation of metastases remains unclear. Several reports have indicated an association between UCHL1 expression levels in tumor tissues and prognosis or tumor progression [17,19,20,22,27,28]. However, there are few reports on the association between serum UCHL1 levels and tumor progression. Research on breast cancer suggested that serum UCHL1 that is transported by extracellular exosomes may be involved in drug resistance. Furthermore, this study shows that UCHL1 levels in tissue and serum are positively correlated [38]. Serum UCHL1 levels in EC patients may also be regulated through a mechanism similar to that described in this report. Cancer cells released from primary tumors, known as circulating tumor cells (CTCs), play a key role in metastatic lesion formation. CTCs have been reported to be associated with metastasis through the EMT [39]. This observation may also help clarify the potential association between serum UCHL1 and metastasis.

This study has several limitations. First, the role of UCHL1 in regulating EMT-related molecules remains unclear. Since the mechanisms promoting EMT are complex and involve numerous molecules and signaling pathways, it is necessary to determine which EMT-related molecules are affected by UCHL1. Furthermore, whether the effects of UCHL1 on EMT-related molecules are mediated by its deubiquitination activity or other mechanisms (such as ligase activity) should be determined. In addition, the functional analyses performed in this study used a limited number of endometrial cancer cell lines; analysis with several other EC cell lines would strengthen our understanding of UCHL1’s functional role in EC cell lines. Second, this study did not analyze metastatic regions using animal experiments. To demonstrate that UCHL1 promotes cancer progression in EC by regulating the EMT, evaluation of the metastatic regions in vivo is required. The efficacy and safety of the UCHL1 inhibitor should be demonstrated in vivo to explore its potential as a novel therapeutic agent.

Our results showed a partial role of UCHL1 in EC cells; however, further studies are needed to clarify the association between UCHL1 and EC progression.

## 5. Conclusions

Based on this study, we suggest that UCHL1 regulates the migration and invasion of EC cells in vitro. Furthermore, UCHL1 may promote the cadherin switch through modulation of EMT transcriptional factors. These findings indicate that UCHL1 could be involved in the progression of EC. Further mechanistic and in vivo analyses are warranted to elucidate the detailed functions of UCHL1 in EC progression.

## Figures and Tables

**Figure 1:**
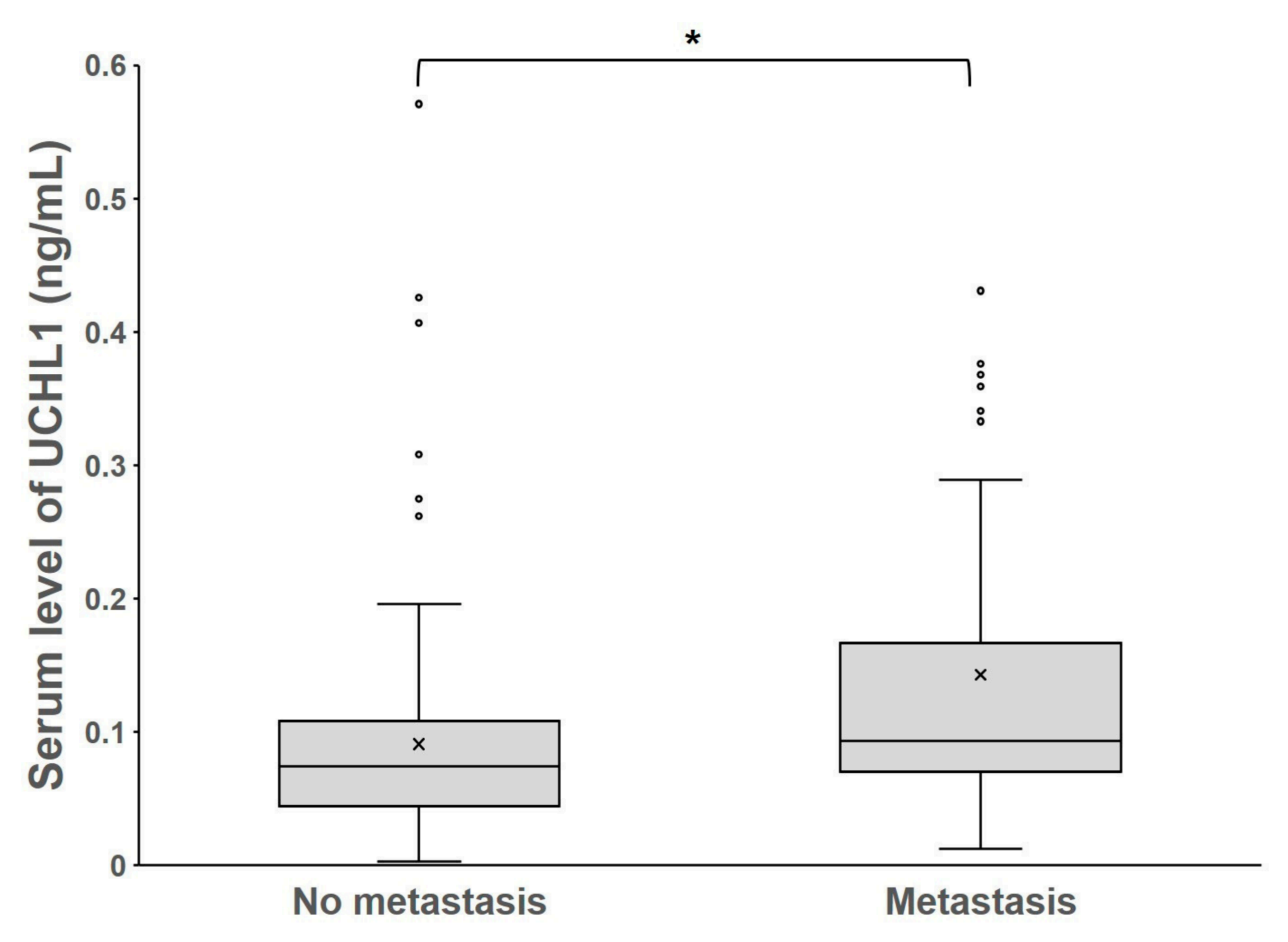
Serum UCHL1 level increased in serum of patients with metastasis. Sera of 149 patients with EC were sampled before surgery. Serum UCHL1 levels were analyzed using a Human UCHL1-ELISA kit and compared based on the presence or absence of metastasis. The metastasis group included lymph node and distant metastases. No metastasis: *n* = 106, metastasis: *n* = 43. Values are presented as medians. Statistical significance was determined using the Mann–Whitney U test. *p* value: * *p* < 0.05.

**Figure 2:**
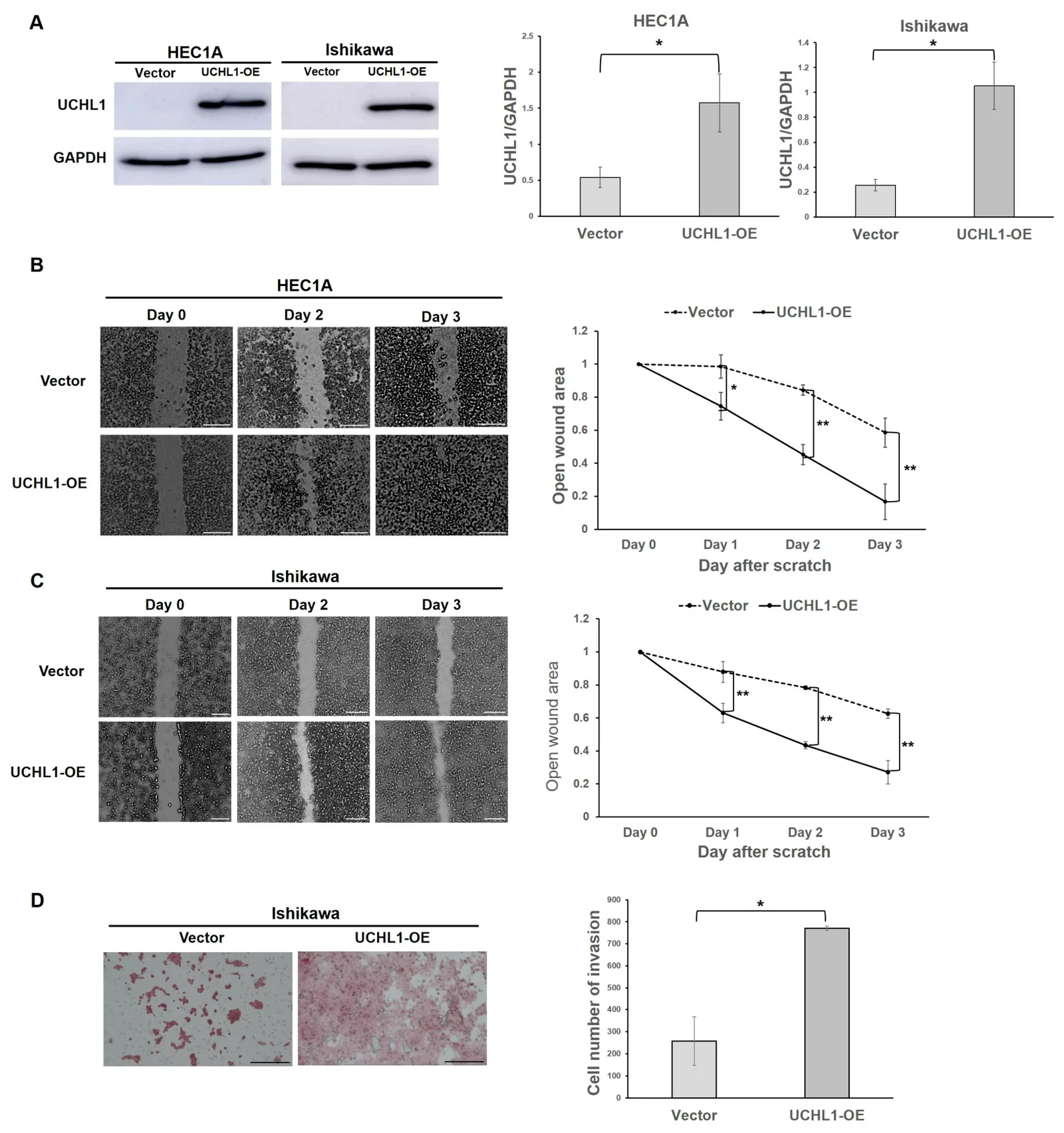
UCHL1 promoted cell migration and invasion in EC cells. (**A**) UCHL1-expression vectors were transfected into EC cell lines (HEC1A and Ishikawa) and UCHL1-OE cell lines were established (UCHL1-OE). UCHL1 protein expression was confirmed using Western blotting. Cell lines transfected with the empty vectors were used as controls (Vector). Wound-healing assays of (**B**) UCHL1-OE HEC1A cells and (**C**) Ishikawa cells. Open wound areas were observed every day and calculated as a ratio relative to the area on day 0. Scale bar is 500 µM. (**D**) Transwell assay of UCHL1-OE Ishikawa cells. After 16 h incubation, the numbers of cells that had infiltrated the surface of lower chamber were counted. Scale bar is 200 µM. All values are expressed as mean ± SD of three independent experiments. Statistical significance was determined using Student’s *t*-test. *p* value: * *p* < 0.05, ** *p* < 0.01.

**Figure 3:**
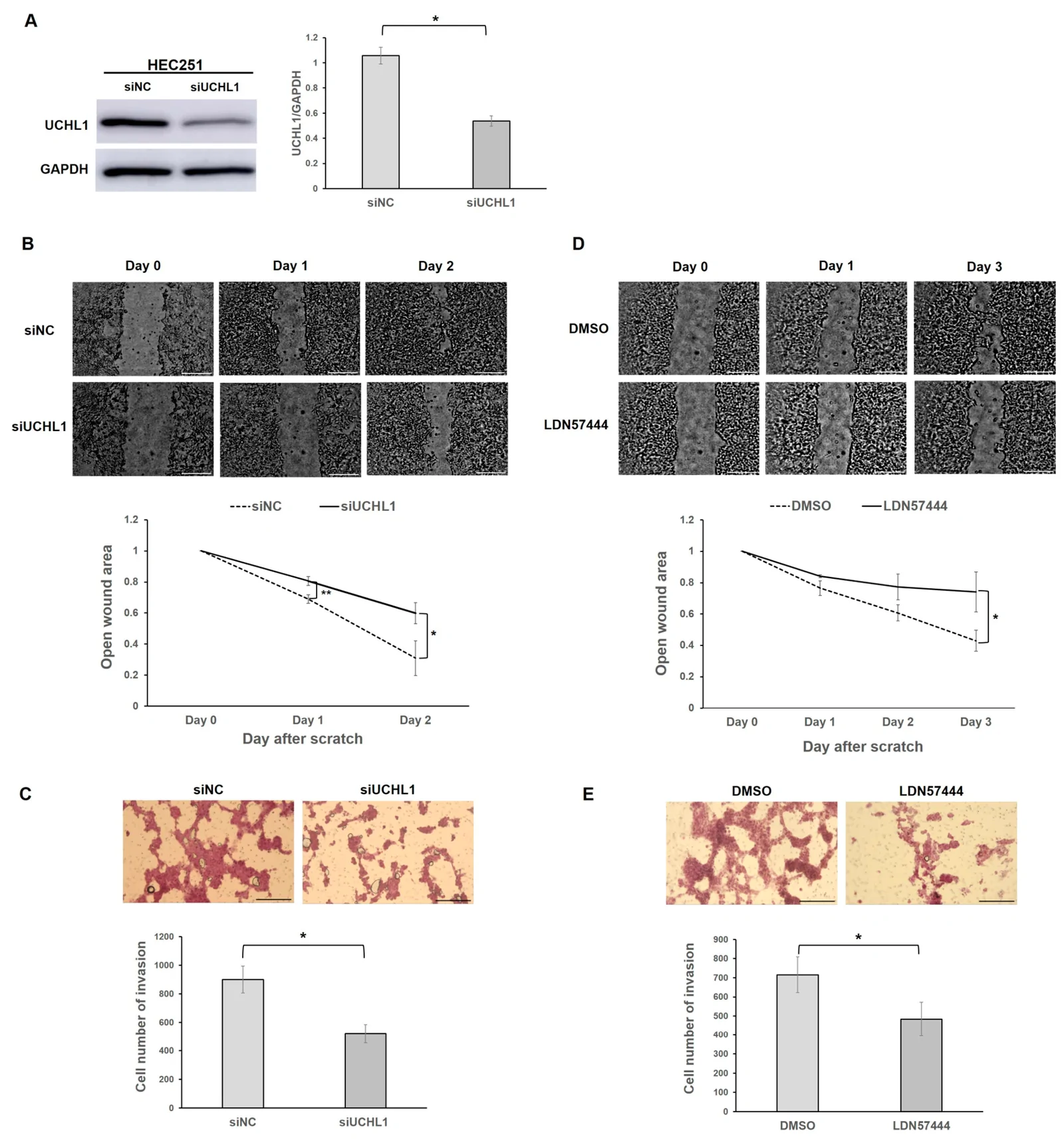
Knockdown or inhibition of UCHL1 suppressed cell migration and invasion in EC cells. (**A**) HEC251 cells were transfected with UCHL1 targeting siRNA (siUCHL1) or a negative control siRNA (siNC). Effective knockdown of UCHL1 was confirmed using Western blotting 72 h after transfection. (**B**) Wound-healing assay of UCHL1-KD HEC251 cells. Open wound areas were observed every day and calculated as a ratio relative to the area on day 0. Scale bar is 500 µM. (**C**) Transwell assay of UCHL1-KD cells. After 16 h incubation, the numbers of cells that had infiltrated the surface of lower chamber were counted. Scale bar is 200 µM. (**D**) Wound-healing and (**E**) transwell assays of HEC251 cells with UCHL1 inhibitor (LDN57444). The scale bars are the same as those of the experiments in KD cells. HEC251 cells treated with DMSO were used as controls. At the start of each experiment, LDN57444 and DMSO were added to HEC251 cells. All values are expressed as the mean ± SD of three independent experiments. Statistical significance was determined using Student’s *t*-test. *p* value: * *p* < 0.05, ** *p* < 0.01.

**Figure 4:**
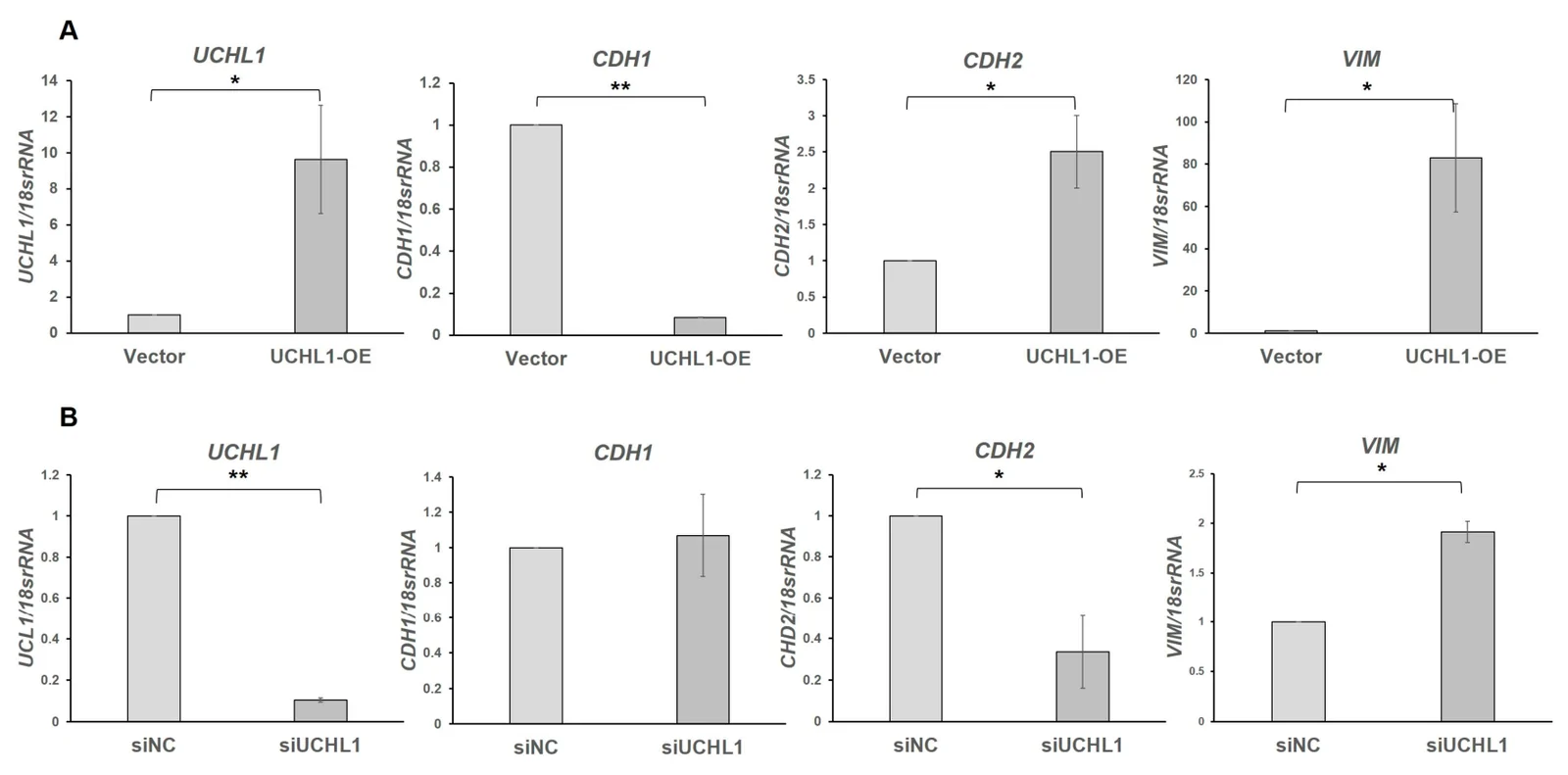
Modulation of UCHL1 altered mRNA expression of EMT-related molecules in EC cells. qRT-PCR was conducted to analyze the mRNA expression of EMT-related molecules in EC cells. CDH1 is the epithelial marker. CDH2 and VIM are mesenchymal markers. The mRNA expressions of EMT-related molecules and UCHL1 in (**A**) UCHL1-OE Ishikawa cells and (**B**) UCHL1-KD HEC251 cells. Relative mRNA levels of the target gene were quantified using the comparative Ct(2^−ΔΔCT^) method. 18srRNA was used as an internal control. Values are expressed as the mean ± SD of three independent experiments. Statistical significance was determined using Student’s *t*-test. *p* value: * *p* < 0.05, ** *p* < 0.01.

**Figure 5:**
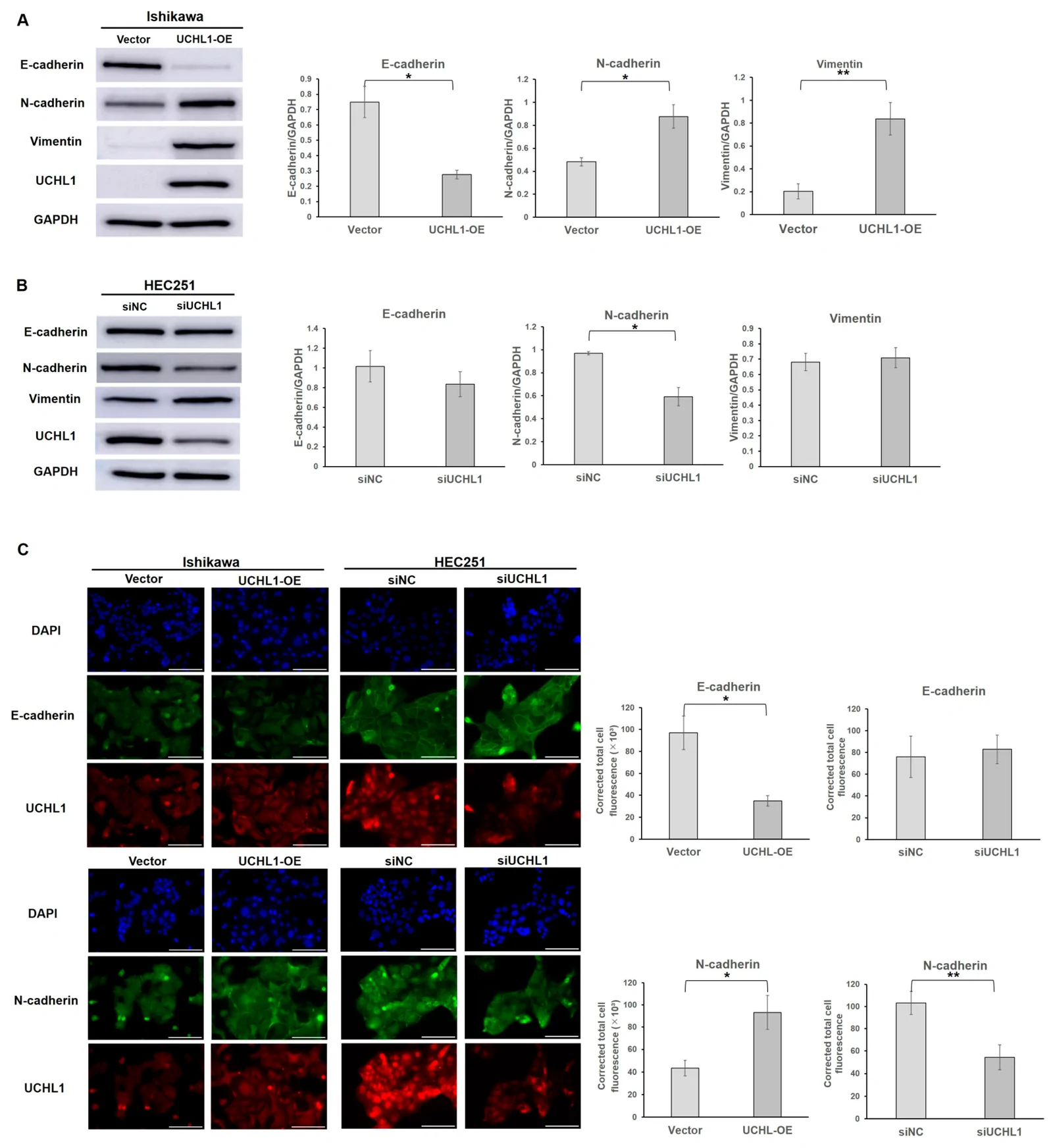
Modulation of UCHL1 altered the expressions of EMT-related proteins in EC cells. The protein expressions of EMT-related molecules were detected using Western blotting and IFA of UCHL1-OE and UCHL1-KD cells. E-cadherin is the epithelial marker. N-cadherin and Vimentin are mesenchymal markers. The protein expression of EMT-related molecules was analyzed using Western blotting in (**A**) UCHL1-OE Ishikawa cells and (**B**) UCHL1-KD HEC251 cells. Values are expressed as the mean ± SD of three independent experiments. (**C**) The protein expression of E-cadherin and N-cadherin was detected using IFA in UCHL1-OE Ishikawa cells and UCHL1-KD HEC251 cells. The same results were confirmed through three independent experiments. Scale bar is 100 µm. The statistical significances of Western blotting and IFA were determined using Student’s *t*-test. *p* value: * *p* < 0.05, ** *p* < 0.01.

**Figure 6:**
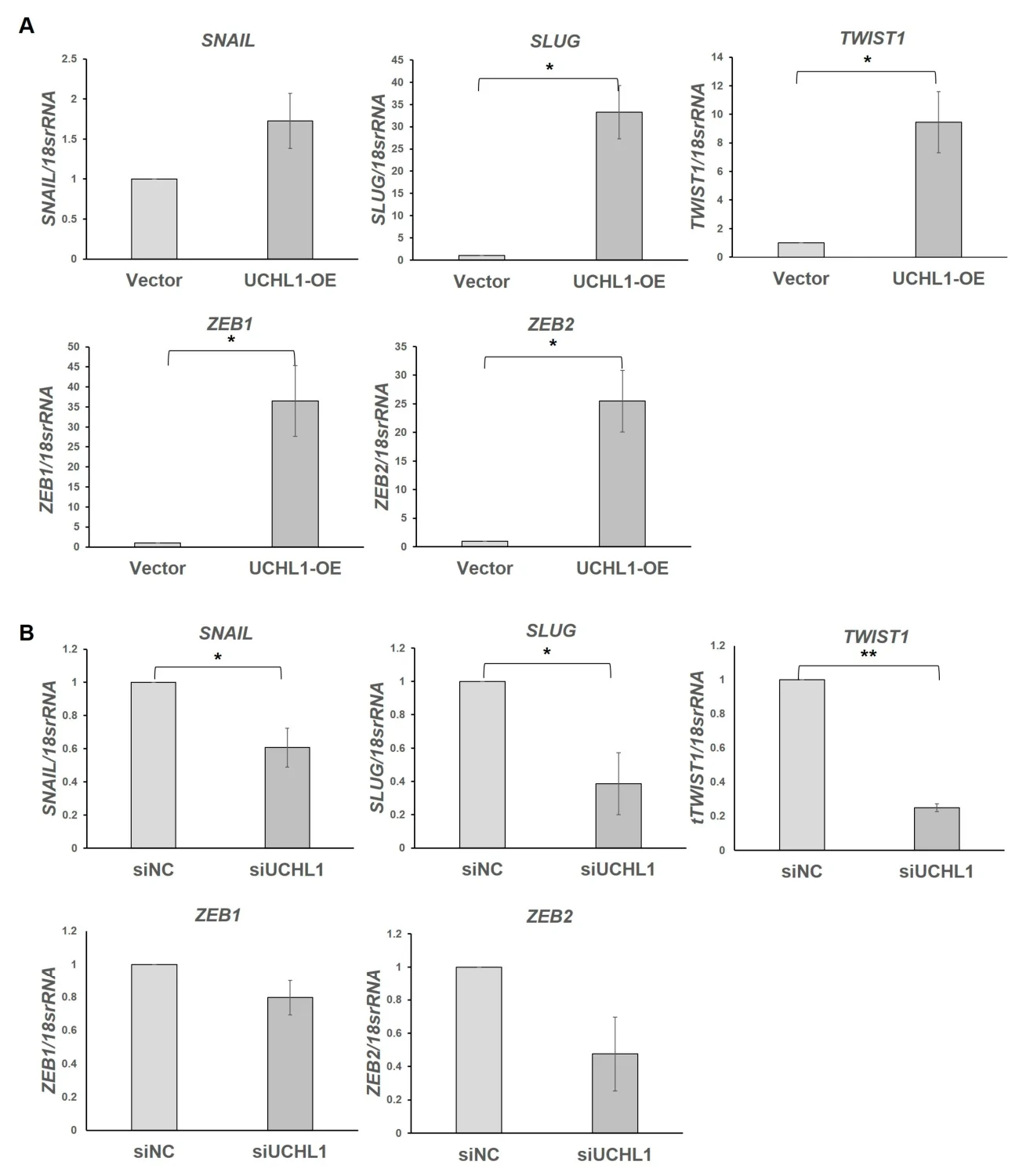
Modulation of UCHL1 altered mRNA expressions of EMT-inducing transcriptional factors (EMT-TFs) in EC cells. qRT-PCR was conducted to analyze the mRNA expression of EMT-TFs in EC cells. mRNA expressions of EMT-TFs in (**A**) UCHL1-OE Ishikawa cells, and (**B**) UCHL1-KD HEC251 cells. Relative mRNA levels of target gene were quantified using the comparative Ct(2^−ΔΔCT^) method. 18srRNA was used as an internal control. Values are expressed as the mean ± SD of three independent experiments. Statistical significance was determined using Student’s *t*-test. *p* value: * *p* < 0.05, ** *p* < 0.01.

## Data Availability

The original contributions presented in this study are included in the article and Supplementary Materials. Further inquiries can be directed to the corresponding author.

## Supplementary Materials

The following supporting information can be downloaded at: https://www.mdpi.com/article/10.3390/biomedicines14102168/s1, Figure S1: UCHL1 protein expression in endometrial cancer cell lines; Figure S2: Proliferation assays of UCHL1-OE cells; Figure S3: Proliferation assay of UCHL1-KD cells; Figure S4: mRNA expressions of *CDH1* and *VIM* in UCHL1-KD cells with an extended knockdown time; Table S1: The sequences of siRNA; Table S2: The sequences of primers for qRT-PCR.

## Abbreviations

The following abbreviations are used in this manuscript:

EC	Endometrial cancer
UCHL1	Ubiquitin C-terminal hydrolase-L1
ELISA	Enzyme-linked immunosorbent assay
UCHL1-OE	UCHL1-overexpressing
UCHL1-KD	UCHL1-knockdown
EMT	Epithelial–mesenchymal transition
DUBs	Deubiquitinating enzymes
qRT-PCR	Real-time polymerase chain reaction
IFA	Immunofluorescence assay
FBS,	Fetal bovine serum
siRNA,	Small interfering RNA
DMSO	Dimethyl sulfoxide
SDS,	Sodium dodecyl sulfate
EMT-TFs	EMT-inducing transcription factors
VEGFA	Vascular endothelial growth factor A
EGFR	Epidermal growth factor receptor
