# Supplementary material for: UCHL1 Promotes Epithelial–Mesenchymal Transition Through E-Cadherin and N-Cadherin Switch in Endometrial Cancer
Source: Biomedicines. 2026 Sep 24;14(10):2168. doi: 10.3390/biomedicines14102168 (PMC13701699; doi:10.3390/biomedicines14102168)
Supplement: Supplementary file 1 [file biomedicines-14-02168-s001.zip › biomedicines-4391247- Supplementary Table S1 and S2.pdf]

Table S1: The sequences of siRNA

siRNA		Sequence, 5-3′
siUCHL1 #1	guide	ACACUUUAGGACUAACUUCUU
	passenger	GAAGUUAGUCCUAAAGUGUAC
siUCHL1 #2	guide	UGAAUAAGUCCGAUUGUGCCA
	passenger	GCACAAUCGGACUUAUUCACG
Negative control	guide	ACUGAUUAUCGAUCUUGCUUC
	passenger	AGCAAGAUCGAUAAUCAGUUU

TableS2: The sequences of primers for qRT-PCR

Gene		Primer sequence, 5'-3'
UCHL1	F	GGAAGGCCAATGTCGGGTAGA
	R	CTTCTCCTTGCTCACGCTCG
CDH1	F	TACCCTGGTGGTTCAAGCTG
	R	CCTGACCCTTGTACGTGGTG
CDH2	F	ATTGCCATCCTGCTCTGCAT
	R	TGGCTCAAGTCATAGTCCTGG
Vimentin	F	TGGACCAGCTAACCAACGAC
	R	ACGCATTGTCAACATCCTGTC
SNAIL	F	TTCTCTAGGCCCTGGCTGCTACAA
	R	TCTTGACATCTGAGTGGGTCTGGA
SLUG	F	CTGGTCAAGAAGCATTTCAACGCC
	R	AAAGAGGAGAGAGGCCATTGGGTA
TWIST1	F	GGAGTCCGCAGTCTTACGAG
	R	TCTGGAGGACCTGGTAGAGG
ZEB1	F	TTCACAGTGGAGAGAAGCCA
	R	GCCTGGTGATGCTGAAAGAG
ZEB2	F	ATAAGGGAGGGTGGAGTGGA
	R	CGCGTTCCTCCAGTTTTCTT
18srRNA	F	GTAACCCGTTGAACCCCATT
	R	CCATCCAATCGGTAGTAGCG

F, forward; R, reverse.
